# College of Pharmacy

**Published:** 1859-12

**Authors:** 


### COLLEGE OF PHARMACY.

An organization under this name has been effected in Chicago. Among the appointments, those of Prof. Blaney to the chair of Chemistry, and F. Scammon to that of Pharmacy, may be noticed as excellent. Similar attempts in other cities have, with few exceptions, failed, but we trust that this may be more fortunate.

